# Bouveret Syndrome: Etiology, Clinical Presentation, Differential Diagnosis, Complications, and Treatment Options

**DOI:** 10.7759/cureus.64754

**Published:** 2024-07-17

**Authors:** Nikhil Thatipalli, Rajesh Gattani, Krushank Nayak, Kesav Sudabattula

**Affiliations:** 1 General Surgery, Jawaharlal Nehru Medical College, Datta Meghe Institute of Higher Education and Research, Wardha, IND

**Keywords:** gallbladder disease, fistula repair, cholecystectomy, surgical treatment, endoscopic intervention, cholelithiasis, bilioduodenal fistula, gallstone ileus, gastric outlet obstruction, bouveret syndrome

## Abstract

Bouveret syndrome is one of the complications of gallstone disease possibly fatal, which proposes the presence of a large stone obliterating the lumen of the duodenum or stomach because of the formation of a bilioenteric fistula. This review article, therefore, plans to review the causes, patient characteristics, diagnostic workup, associated conditions, and treatment of Bouveret syndrome. A literature search was also performed through scientific databases such as Scopus, Google Scholar, and PubMed concerning articles related to Bouveret syndrome written by different authors. The terms employed for the search were bilioduodenal fistula, Bouveret syndrome, gastric outlet obstruction, and gallstone ileus. Both case reports and systematic reviews that were written in the English language and published between the years 2000 and 2024 were considered.

Finally, the review establishes the relevant concerns surrounding the diagnosis of Bouveret syndrome, focusing on the diagnosing issues. It emphasises the need for some specialities' involvement and focuses on the importance of endoscopic intervention. For patients, endoscopy remains the first line of treatment, while surgery is necessary in cases where conservative methods cannot be used. The article also focuses on new approaches to treating the conditions, such as percutaneous gallbladder stone dissolution. Latterly, further developments in minimally invasive surgery pertain to refining methods, including endoscopic removal and lithotripsy, to improve the survival rate of patients. Further investigation is required, especially regarding the administration schedule in relation to this disorder and goals that can reduce mortality and morbidity, especially in elderly patients with comorbid diseases.

## Introduction and background

Gastric outlet obstruction is the trademark of the exceptional gallstone disease condition known as Bouveret syndrome [1]. This uncommon gallstone ileus is caused by a vast stone impaction within the proximal duodenum or pylorus due to an unconstrained fistula between the stomach or duodenum and the gallbladder [2]. M. Beassier, a French specialist, was the primary to depict it preoperatively in 1770 [3]. Afterwards, this sickness was called for by the French doctor L. Bouveret, who distributed two careful case reports of it in 1896 [1,4]. The critical passing rate related to Bouveret syndrome, generally 12-30%, is ascribed to the disease's complexity, non-specific presentation, and tendency to influence older adults [5]. Besides, there's no agreement around the diagnostic workup and administration counting endoscopy, laparoscopy, and open surgery due to the disease's rarity [6]. An enormous stone that blocks the stomach outflow and voyages through a bilioduodenal fistula is the cause of Bouveret syndrome [7]. Due to non-specific indications and laboratory results, there's a significant hazard of misdiagnosing Bouveret syndrome [4,8]. Bouveret syndrome is the foremost exceptional kind of gallstone ileus [7]. It happens when a gallstone relocates using a bilioenteric fistula, obstructing the gastric outlet and, in uncommon events, other parts of the digestive tract [8,9]. The entrance location is, as a rule, a fistula between the gallbladder and a segment of the stomach or digestive system [10]. A history of cholelithiasis, stones larger than 2-8 cm, female, and older than 60 are among the risk factors [1,7]. Key characteristics of Bouveret syndrome are described in Table 1.

**Table 1:** Characteristics of Bouveret syndrome Table created by Nikhil Thatipalli References: [1-9]

Feature	Description
Type of gallstone disease	Uncommon gallstone ileus
Cause	Large gallstone impaction in the duodenum or pylorus due to fistula between the stomach/duodenum and gallbladder
First described by	M. Beassier (1770, preoperatively)
Named after	L. Bouveret (1896)
Mortality rate	12-30% (attributed to complexity, non-specific symptoms, and older patient population)
Diagnosis	No single agreed-upon method (may involve endoscopy, laparoscopy, or open surgery)
Symptoms	Non-specific (nausea, vomiting, abdominal pain, distention)
Risk factors	History of gallstones, large stones (>2-3 cm), female gender, age >60
Mechanism	Gallstone travels through bilioduodenal fistula, blocking the stomach outlet
Location of fistula	Usually between the gallbladder and stomach/duodenum

## Review

Search methodology

**Figure 1:**
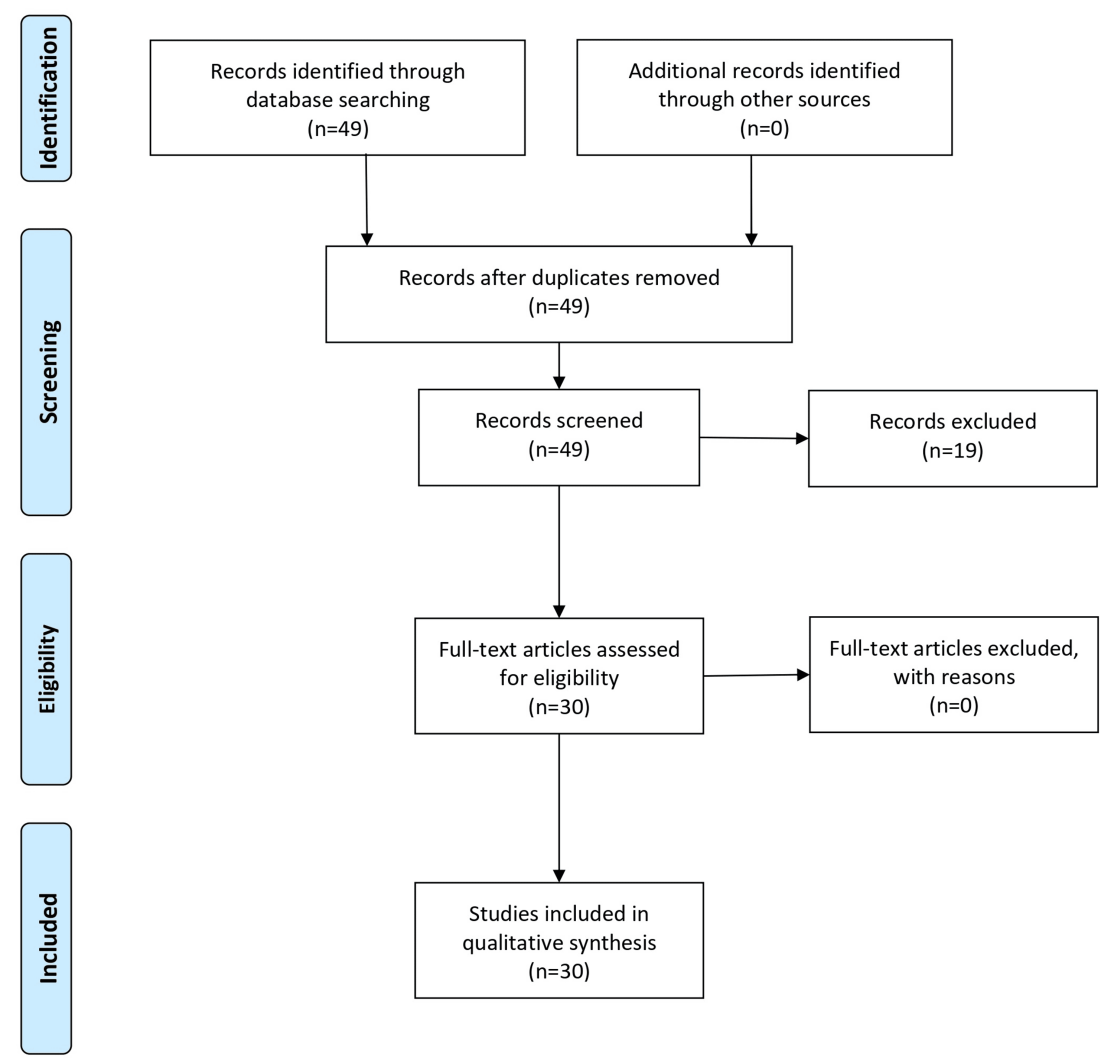
PRISMA flow diagram Figure created by Nikhil Thatipalli PRISMA: Preferred Reporting Items for Systematic Reviews and Meta-Analyses

A literature search was conducted for this review of Bouveret syndrome. The search was conducted using databases such as Scopus, Google Scholar, and PubMed. The MeSH terms and a few keywords used were bilioduodenal fistula, Bouveret syndrome, gastric outlet obstruction, and gallstone ileus. The articles were chosen for their relevance, timeliness, and availability of clinical information, pathophysiology, differential diagnosis, complications, and available treatments. Only peer-reviewed English-language articles were included, such as case studies and systematic reviews published between 2000 and 2024. The Preferred Reporting Items for Systematic Reviews and Meta-Analyses (PRISMA) flow diagram is shown in Figure 1.

Pathophysiology of Bouveret syndrome

Bouveret syndrome may be an exceptionally exceptional condition that accounts for 0.3-5% of complications from cholelithiasis and accounts for 1-3% of all gastrointestinal (GI) tract blockages brought on by gallstones [11]. However, most stones are small and either pass undetected or have terminal ileum impaction [12]. Inflammation and attachment of the gallbladder to the GI system may ensue from an acute cholecystitis episode [13]. This can cause an ischemia tear of the adjacent gallbladder and enteric wall in addition to the mechanical strain that gallstones put on the gallbladder and the bowel wall [14]. Gallstone passage is facilitated by this process, which intensifies the development of a fistula between the gallbladder and the colon [15].

In addition to acute cholecystitis, a few case reports resulting from gallbladder malignancy have been released, describing the development of a cholecystoenteric fistula [3,10,16]. Comprising 68% of cases, bilioenteric fistulas typically manifest as cholecystoduodenal fistulas [17]. Cholecystocolic and cholecystogastric fistulas are less frequent variations that comprise 17% and 5% of cases, respectively [17]. Due to the thickness of the stomach wall, cholecystogastric fistulas are most likely the rarest [18]. Even with a bilioenteric fistula, most ectopic gallstones are removed by vomiting or diarrhea [9]. In addition to other variables, such as pre-existing stenosis or changed GI tract anatomy following surgery, more giant stones are likely to cause clinical signs of gastric outlet obstruction [19]. Clinical features of Bouveret syndrome are described in Table 2.

**Table 2:** Clinical features of Bouveret syndrome Table created by Nikhil Thatipalli References: [9,11-17,19] GI: gastrointestinal

Feature	Description
Incidence in cholelithiasis complications	0.3-5%
Incidence in GI blockages	1-3%
Usual stone size	Small, undetected, or passed through the terminal ileum
Cause	Acute cholecystitis episode leading to inflammation and attachment to the GI tract
Pathogenesis	
Mechanical strain	Gallstones put pressure on the gallbladder and bowel wall
Ischemia tear	Inflammation weakens adjacent tissue causing tears in the gallbladder and intestinal wall
Fistula development	Tear facilitates gallstone passage and fistula formation between the gallbladder and colon
Other causes	Gallbladder malignancy (rare)
Most common bilioenteric fistula	Cholecystoduodenal fistula (68% cases)
Less common fistulas	Cholecystocolic (17% cases) and cholecystogastric (5% cases)
Reason for rarest gastric fistula	Thick stomach wall
Ectopic gallstone passage	Vomiting or diarrhea (even with bilioenteric fistula)
Clinical signs of gastric outlet obstruction	Larger stones, pre-existing stenosis, or altered GI anatomy post-surgery

Differential diagnosis of Bouveret syndrome

Differential diagnosis of Bouveret syndrome includes a wide range of illnesses. A duodenal web, or membrane-like blockage within the duodenum, is one of the congenital causes [10]. Erosive gastritis, which causes erosion and inflammation of the stomach lining, peptic ulcer disease, which is characterized by sores in the stomach or duodenal lining, and Crohn's disease, an inflammatory bowel disease that can affect any part of the GI tract, are examples of conditions that have an inflammatory cause [12,20]. Multiple forms of cancer are among the malignant causes of Bouveret syndrome. Duodenal carcinoma develops in the duodenum, whereas gastric antral carcinoma begins in the stomach's antrum [21]. Other potential malignant causes include ampullary carcinoma, which appears at the ampulla of Vater, the point where the bile and pancreatic ducts converge, and pancreatic carcinoma, a cancer of the pancreas [12,22]. A further possible malignant cause of this illness is cholangiocarcinoma, often known as bile duct cancer [16,23]. Every one of these ailments has the potential to cause a bilioduodenal fistula, which in turn exacerbates Bouveret syndrome [1,10]. Differential diagnosis of Bouveret syndrome is shown in Table 3.

**Table 3:** Differential diagnosis of Bouveret syndrome Table created by Nikhil Thatipalli References: [1,10,12,16,21-23] GI: gastrointestinal

Category	Condition	Description
Congenital	Duodenal web	Membrane-like blockage within the duodenum
Inflammatory	Erosive gastritis	Erosion and inflammation of the stomach lining
	Peptic ulcer disease	Sores in the stomach or duodenal lining
	Crohn's disease	Inflammatory bowel disease affecting any part of the GI tract
Malignant	Duodenal carcinoma	Cancer developing in the duodenum
	Gastric antral carcinoma	Cancer starting in the stomach's antrum
	Ampullary carcinoma	Cancer at the ampulla of Vater (junction of the bile and pancreatic ducts)
	Pancreatic carcinoma	Cancer of the pancreas
	Cholangiocarcinoma	Bile duct cancer

Complications and treatment of Bouveret syndrome

Untreated Bouveret syndrome can lead to persistent blockage of the gastric outlet, which can cause anorexia, dehydration, malnutrition, and abnormalities in electrolytes [2,17,24]. Intestinal perforation is the most dangerous consequence since it can cause severe morbidities [25]. Recurrent Bouveret syndrome, biliary infection, gallstone pancreatitis, and maybe even cancer are risks associated with leaving a fistula untreated [12,26]. There is always a danger of bleeding and infection during surgery [23]. Furthermore, the biliary tree may unintentionally be harmed by cholecystectomy, mainly if inflammation is present [4]. Complications of Bouveret syndrome are mentioned in Table 4.

**Table 4:** Complications of Bouveret syndrome Table created by Nikhil Thatipalli References: [2,4,12,17,24-26]

Complication	Description
Persistent blockage	Blockage of the gastric outlet can lead to anorexia, dehydration, malnutrition, and electrolyte imbalances
Intestinal perforation	The most dangerous complication, causing serious health problems
Recurrent Bouveret syndrome	A fistula left untreated increases the risk of recurrence
Biliary infection	Untreated fistula can lead to biliary infection
Gallstone pancreatitis	Gallstones can migrate and cause pancreatitis
Cancer (potential)	Untreated fistula may be a risk factor for cancer (more research needed)
Surgical risks	Bleeding and infection are potential risks during surgery
Biliary tree injury	Cholecystectomy, especially with inflammation, may unintentionally damage the biliary tree

Currently, the first line of treatment for Bouveret syndrome is endoscopic intervention [27]. Endoscopic retrieval is one form of minimally invasive therapy, along with mechanical, electrohydraulic lithotripsy, laser, and extracorporeal shock waves [27,28]. When an endoscopic attempt fails, or no technical knowledge is available, surgical solutions may need to be explored in treating individuals with Bouveret syndrome [4,27]. The surgical strategy involves performing an open gastrotomy, pylorotomy, or duodenotomy at or near the obstruction location [25,29]. If moving an affected duodenal gallstone into the stomach is feasible, a gastrostomy may remove the stone [4]. It's controversial if treating Bouveret syndrome requires a multi-step procedure that combines cholecystectomy and fistula repair [30]. Due to their age and numerous comorbidities, the majority of patients are not good candidates for surgery [30]. The treatment of Bouveret syndrome is shown in Table 5.

**Table 5:** Treatment of Bouveret syndrome Table created by Nikhil Thatipalli References: [4,25,27-30]

Treatment	Description
Endoscopic intervention (preferred)	First-line treatment. Includes endoscopic retrieval, mechanical lithotripsy, electrohydraulic lithotripsy, laser lithotripsy, and extracorporeal shock wave lithotripsy
Surgery (if endoscopy fails)	Options include open gastrotomy, pylorotomy, or duodenotomy. Gastrostomy may be used in specific situations
Cholecystectomy and fistula repair (controversial)	May be part of a multi-step procedure, but suitability depends on individual health factors

The future of Bouveret syndrome treatment

Nevertheless, Bouveret syndrome treatment is transitional from typical open surgeries [4]. Surgical methods are now being supplemented by endoscopic techniques, which can require fewer invasions of the body and prove to be more advantageous to the patients [31]. Electrohydraulic lithotripsy, for instance, applies electrical energy to break up the obstructing gallstone within the duodenum so that it can pass through easily [31]. However, it is accompanied by the disadvantage of causing some harm to the neighbouring tissues [31]. The researchers are working closely to fine-tune such methods to enhance more efficient fragmentation of the stones and reduce the undesired implications [32]. Another potential method is endoscopic retrieval through baskets and nets [32]. The endoscopic retrieval approach needs particular dexterity to eliminate the stone completely [32]. Nevertheless, researchers are investigating more developed instruments in terms of grasping and visualization modules to raise the success ratios of endoscopic retrieval [32].

Besides such developments, further treatment strategies for Bouveret syndrome might include percutaneous gallbladder stone dissolution [5,22]. This method employs drugs that are applied topically on the skin and directly to the organ, which is the gallbladder, to dissolve the stones without surgery [22]. Although its use in the management of Bouveret syndrome is still under research, percutaneous dissolution can eradicate the syndrome completely because it deals with the cause of the syndrome, which is gallstones, through a non-surgical technique [31,32]. Studies included in the article are mentioned in Table 6.

**Table 6:** Studies included in the article A table was created by the author to analyze the included studies since we have used a systematic review approach search methodology Table created by Nikhil Thatipalli

Study reference	Year	Study type	Key findings	Treatment approaches	Outcomes
Haddad et al. [1]	2018	Literature review	Review of existing literature on Bouveret syndrome	Various including endoscopic and surgical	Varied outcomes, often good with appropriate treatment
Philipose et al. [2]	2019	Case report	Detailed case of Bouveret syndrome	Endoscopic management	Successful outcome
Ramos and Chiang [3]	2018	Case report	Description of a case with diagnostic imaging	Endoscopic retrieval	Successful outcome
Koulaouzidis and Moschos [4]	2007	Narrative review	Comprehensive review of Bouveret syndrome	Various	Varied outcomes
Al-Habbal et al. [5]	2017	Case report and systematic review	Case report and systematic review of Bouveret syndrome	Endoscopic and surgical	Varied outcomes, often positive with intervention
Doody et al. [6]	2007	Case report	Variant presentation of Bouveret syndrome	Endoscopic intervention	Successful outcome
Yu et al. [7]	2019	Case report	Rare presentation causing gastric outlet obstruction	Surgical treatment	Successful outcome
Castro et al. [8]	2008	Case report	Case report of Bouveret syndrome	Surgical management	Successful outcome
Satchithanandha et al. [9]	2023	Case report	Two different treatment approaches for the same condition	Endoscopic and surgical	Successful outcomes
Turner et al. [10]	2024	Case report	Bouveret syndrome with bilioduodenal fistula	Surgical intervention	Successful outcome
Valgaeren et al. [11]	2023	Case report	Gastric outlet obstruction due to complicated cholelithiasis	Endoscopic treatment	Successful outcome
Runyan et al. [12]	2021	Case series	Series of cases illustrating complications	Endoscopic and surgical treatments	Varied, generally positive
Chen et al. [13]	2020	Case report	Complicated case with acute cholecystitis	Surgical management	Successful outcome
Frąk et al. [14]	2022	Case report	Rare variant of ileus	Surgical intervention	Successful outcome
Santos-Rancaño et al. [15]	2016	Case report	Detailed case of Bouveret syndrome	Endoscopic treatment	Successful outcome
Rey Chaves et al. [16]	2022	Case report	Cholecystogastric fistula in Bouveret syndrome	Surgical management	Successful outcome
Mavroeidis et al. [17]	2013	Case report	Case report with literature review	Surgical intervention	Successful outcome
Osman et al. [18]	2020	Case report	Bouveret syndrome with cholecystogastric fistula	Surgical management	Successful outcome
Iancu et al. [19]	2008	Case report	Bouveret syndrome with acute gangrenous cholecystitis	Surgical treatment	Successful outcome
Ha et al. [20]	2004	Case report	Case of pseudo-Bouveret syndrome	Endoscopic intervention	Successful outcome
Sharma et al. [21]	2010	Case report	Carcinoma gallbladder with Bouveret syndrome	Surgical treatment	Successful outcome
Lopes et al. [22]	2017	Case report	Bouveret syndrome with pancreatic acinar cell carcinoma	Surgical intervention	Successful outcome
Adnan et al. [23]	2022	Case series	Series of Bouveret syndrome cases	Various treatments	Varied outcomes, often positive
Jin and Naidu [24]	2021	Case report	Rare form of gastric outlet obstruction	Endoscopic treatment	Successful outcome
Sabir et al. [25]	2024	Case report	Rare case of gallstone causing gastric outlet obstruction	Endoscopic treatment	Successful outcome
Wang et al. [26]	2019	Case report	Case report of Bouveret syndrome	Endoscopic treatment	Successful outcome
Sanyang et al. [27]	2022	Case report	Successful endoscopic management	Endoscopic treatment	Successful outcome
Avci et al. [28]	2019	Case report	Bouveret syndrome treated with electrohydraulic lithotripsy	Endoscopic electrohydraulic lithotripsy	Successful outcome
Hanandeh et al. [29]	2019	Case report	Surgical duodenotomy following untreated Bouveret syndrome	Surgical duodenotomy	Successful outcome
Taggarsi et al. [30]	2021	Case report	Strategic approach for the management of Bouveret syndrome	Combined endoscopic and surgical strategies	Successful outcome
Troncone et al. [31]	2022	Comprehensive review	Review of new and old lithotripsy techniques for difficult biliary stones	Various lithotripsy techniques	Varied, generally positive
Konuma et al. [32]	2011	Case report	Endoscopic retrieval of a gastric trichobezoar	Endoscopic retrieval	Successful outcome

## Conclusions

The uncommon variant of gallstone ileus known as Bouveret syndrome has a high morbidity rate and vague symptoms that make it difficult to diagnose and treat. A multidisciplinary approach is necessary for effective care, with endoscopic intervention as the primary therapeutic option. When endoscopic techniques don't work, surgery becomes an alternative. Improving patient outcomes requires an early and precise diagnosis informed by a narrative understanding of the pathophysiology and clinical symptoms. Standardized diagnostic and treatment techniques might lower related mortality and morbidity; they could be established with more studies, especially in older patients with many comorbidities.
